# Global Burden and Inequalities in Mental Disorders Across Countries and Territories: 1990 to 2021

**DOI:** 10.31083/AP51756

**Published:** 2026-08-13

**Authors:** Dongjun Zhang, Jiali Wu, Mingyue Wu, Xin Chen, Mengyao Jin, Jiahan Gao, Chenglin Zhang, Lina Wang

**Affiliations:** ^1^School of Psychology, Henan Medical University, 453003 Xinxiang, Henan, China; ^2^School of Health Management, Henan Medical University, 453003 Xinxiang, Henan, China; ^3^School of Nursing, Henan Medical University, 453003 Xinxiang, Henan, China

**Keywords:** mental disorders, regional inequality, disability-adjusted life-years, global burden of disease study

## Abstract

**Background:**

Although the global burden of mental disorders has been extensively documented, systematic assessments of cross-country inequalities, particularly regarding how this burden varies across levels of socio-demographic development, remain limited.

**Methods:**

Data on disability-adjusted life-years (DALYs) attributable to mental disorders and the socio-demographic index (SDI) were obtained from the 2021 Global Burden of Disease Study. Absolute and relative inequalities were assessed using the slope index of inequality (SII) and concentration index (CI).

**Results:**

Between 1990 and 2021, the total number of DALYs attributable to mental disorders increased by 73.4%, with the largest increase observed among adults aged 30 to 54 years. Age-standardized DALY rates increased by 9.4%, primarily driven by anxiety disorders, eating disorders, and depressive disorders. Females exhibited higher age-standardized rates of depressive disorders, anxiety disorders, bipolar disorder, and eating disorders, whereas males had higher rates of schizophrenia, autism spectrum disorder, conduct disorder, attention-deficit/hyperactivity disorder (ADHD), and other mental disorders. Anxiety disorders and depressive disorders were the primary contributors to the increasing burden, particularly among women and adolescents. In 2021, the burden of mental disorders showed substantial geographical variation across countries and territories. Inequality analyses demonstrated that the SII for overall mental disorders increased from 69 to 243, whereas relative inequality remained stable over time. Across SDI strata, high-SDI countries experienced a greater burden of eating disorders, ADHD, schizophrenia, and anxiety disorders, while low-SDI countries experienced a greater burden of depressive disorders and idiopathic developmental intellectual disability.

**Conclusions:**

The burden of mental disorders remains substantial and unequally distributed worldwide. Persistent and widening disparities over the past three decades highlight the urgent need for strengthened global health policies and coordinated multilevel interventions aimed at reducing inequalities across countries with differing levels of socio-demographic development.

## Main Points

1. Global burden and rising trends: From 1990 to 2021, the total disability-adjusted life-years due to mental disorders increased by 73.4%, with anxiety and eating disorders showing the fastest growth in age-standardized rates. Females consistently bore a higher burden of depressive, anxiety, bipolar, and eating disorders, whereas males experienced a greater burden of schizophrenia, autism spectrum disorders, conduct disorder, and attention-deficit/hyperactivity disorder (ADHD).

2. Widening inequalities across countries: Absolute inequality for overall mental disorders increased more than three-fold (from 69 to 243), indicating widening gaps between high- and low-socio-demographic index (SDI) countries. Relative inequality remained stable overall (0.01), but disorder-specific trends emerged: increasing for schizophrenia and ADHD, and decreasing for most other disorders. High-SDI countries carried heavier burdens of eating disorders, ADHD, schizophrenia, and anxiety disorders, while low-SDI countries bore heavier burdens of idiopathic developmental intellectual disability and depressive disorders.

3. Policy implications: Persistent and widening disparities in mental disorder burden across levels of socio-demographic development underscore an urgent need for strengthened global health policies. Prioritizing anxiety and depressive disorders—particularly among women, adolescents, and adults aged 30–54 years—while addressing the distinct needs of high-SDI versus low-SDI countries is essential to reduce international inequalities and achieve universal mental health coverage.

## 1. Introduction

Mental disorders affect nearly one billion people worldwide and remain among the top contributors to the global burden of disease [1,2]. They lead to long-term disability, premature mortality, and substantial socioeconomic consequences for individuals, families, and health systems [3,4]. Affected individuals have a life expectancy 10–15 years shorter on average than the general population, with reductions up to 20 years in severe cases [5,6,7,8,9]. Although early intervention and treatment advances have improved outcomes for some conditions, access to mental health services remains uneven across countries [10,11]. Social determinants—including education, income, stigma, and housing—continue to shape mental health outcomes and exacerbate existing disparities [12,13].

Previous studies have used earlier Global Burden of Disease (GBD) data to assess global and regional prevalence of mental disorders and their disability-adjusted life-years (DALYs) burden [14,15]. The GBD 2010 study provided the first comprehensive estimates of mental and substance use disorders, reporting that they accounted for 7.4% of global DALYs and were the leading cause of years lived with disability worldwide [14]. Subsequently, the GBD 2019 Mental Disorders Collaborators found no significant decline in the global age-standardized DALYs rate for mental disorders between 1990 and 2019, with depressive and anxiety disorders remaining the dominant contributors [15]. Several region-specific studies have further expanded the literature. For instance, Liu et al. [16] examined eating disorder trends among adolescents and young adults from 1990 to 2021, highlighting rapid growth in high-income regions. Gowda and Isaac [17] described models of schizophrenia care in community settings across different countries. However, most existing studies have primarily focused on descriptive trends, single disorders, specific regions, or relatively short time frames [5,14,15].

Recently, Zhang et al. [18] used GBD 2021 data to examine trends in depressive and anxiety disorders among adolescents and young adults (aged 10–24 years), employing the slope index of inequality (SII) and concentration index (CI) to assess cross-country inequalities. Although their work provides valuable insights into two major disorders in a vulnerable age group, it does not cover the full spectrum of mental disorders nor assess inequality patterns across all ages. More importantly, no prior study—including Zhang et al. [18]—has quantified the distribution of burden for ten distinct mental disorder subtypes across countries by SDI level, nor incorporated standardized inequality measures (SII for absolute inequality, CI for relative inequality) to assess both dimensions over time [19]. The GBD 2019 study reported DALYs estimates for 12 mental disorders but did not explicitly examine cross-country inequalities using these metrics [15]. Similarly, although GBD 2021 has enabled burden estimates for specific disorders, regions, or age groups [16,20]—including recent studies on subpopulations (e.g., adolescents and young adults [18], children [21]) or single disorders (e.g., bipolar disorder [22])—none has systematically examined both absolute and relative inequalities for all ten mental disorder subtypes across 204 countries over the full 1990–2021 period using dual inequality metrics.

The present study leverages the GBD 2021 dataset, which offers three advantages over earlier releases. First, it extends the observation period to 2021, encompassing two additional years beyond GBD 2019—a period that includes the Coronavirus disease 2019 (COVID-19) pandemic [20]. Second, it incorporates newly available surveys from 2020–2021 with improved data coverage for low- and middle-income countries, enhancing the precision of global burden estimates [23,24]. Third, the SDI has been updated to 2021 levels, ensuring that inequality assessments reflect current socioeconomic development [25]. These enhancements make GBD 2021 uniquely suited for examining long-term trends and inequalities in mental disorder burden across countries at different stages of development.

To address these gaps, we used GBD 2021 data to answer three research questions. First, how did the global burden of ten mental disorder subtypes vary by age, sex, and geography from 1990 to 2021? Second, did absolute and relative cross-national inequalities in mental disorder burden—measured by the SII (on the original DALYs scale) and CI (proportional concentration)—widen or narrow over the 32-year period across countries with different levels of socio-demographic development? Third, which specific mental disorder subtypes showed increasing inequality between high-SDI and low-SDI countries, and which displayed decreasing or stable patterns? By systematically examining these questions using long-term, globally comparable estimates, this study extends previous GBD-based research by offering, to our knowledge, the first simultaneous assessment of both absolute and relative inequalities for ten mental disorder subtypes over a 32-year period. This provides new evidence that goes beyond the disorder- and age-restricted findings of Zhang et al. [18] and other recent analyses, and informs targeted policies aimed at reducing international disparities in mental health.

## 2. Materials and Methods

### 2.1 Data Source

We extracted data from GBD 2021, which provides annual burden estimates for 371 diseases and injuries across 204 countries and territories from 1990 to 2021 [23,24]. For each country and year, we obtained DALYs counts, age-standardized and crude DALYs rates, 95% uncertainty intervals (UIs), and SDI values for all ten specific mental disorder subtypes. These included depressive disorders, bipolar disorder, anxiety disorders, eating disorders, schizophrenia, autism spectrum disorders, attention-deficit/hyperactivity disorder (ADHD), idiopathic developmental intellectual disability (IDID), conduct disorder, and other mental disorders, as defined by GBD 2021 according to Diagnostic and Statistical Manual of Mental Disorders, Fourth Edition (DSM-IV) and the International Classification of Diseases 10, 10th Revision (ICD-10) [24]. The SDI is a composite indicator of development, calculated as the geometric mean of lag-distributed income per capita, average years of schooling, and fertility rate in females under 25 years; values range from 0 (theoretical minimum) to 1 (theoretical maximum) [24,25]. Countries and territories were categorized into SDI quintiles: low (0.00–0.45), low-middle (0.45–0.61), middle (0.61–0.69), high-middle (0.69–0.81), and high (0.81–1.00) [25]. The ten subtypes were selected due to their public health significance and availability in GBD 2021 [20]. The 2020–2021 estimates incorporate newly available epidemiological data, with improved coverage for low- and middle-income countries compared with previous GBD iterations [24].

Data were extracted via the GBD 2021 Results Tool (https://gbd2021.healthdata.org/gbd-results/). For each subtype (excluding substance use disorders, which are reported separately in the GBD framework), age-standardized DALYs rates (per 100,000 population), crude DALYs rates, and total DALYs counts, stratified by sex, 5-year age group (<5 to 95+), year (1990–2021), and location (204 countries and territories), were downloaded. Corresponding SDI values were also obtained. The full GBD 2021 methodology is described elsewhere [24], here we summarize the key aspects relevant to this study.

### 2.2 Burden Description

We performed descriptive analyses to characterize the global burden of the ten mental disorder subtypes. For each subtype, total and age-standardized DALYs in 1990 and 2021, percentage changes over time, and the top and bottom five countries by age-standardized DALYs rates were calculated. Countries were classified into SDI quintiles according to GBD 2021. To examine demographic patterns, we compared age-standardized DALYs rates between sexes for each subtype and visualized age-specific DALYs distributions using population pyramids. Spatial distributions of age-standardized DALYs rates across countries were mapped to identify geographical heterogeneity.

### 2.3 Inequality Analysis

Following World Health Organization recommendations, SII and CI were used to quantify absolute and relative inequalities in mental disorder burden across countries and territories [26]. Our two-time-point approach prioritizes long-term net change and avoids multiple comparison issues; however, it does not capture non-monotonic patterns between 1990 and 2021.

SII was calculated as follows. Countries were ranked by SDI, and each country was assigned a relative rank *R_i_* = (∑*_j_*_<_ *_I_p*_j_ + 0.5*p_i_*)/∑*_j_p_j_*, where *p_i_* is population [26]. A weighted linear regression was then fitted: *Y_i_* = *α* + *βR_i_* + *ε_i_*, where *Y_i_* is the age-standardized DALYs rate and weights are population shares (*w_i_* = *p_i_*/∑*p_j_*) to account for heteroscedasticity [1,26]. The coefficient *β^^^* is the SII, representing the absolute difference in DALYs rates between the most- and least-advantaged countries. Linearity was assessed visually via scatter plots and residual checks; no systematic non-linearity was observed.

CI was computed as the area under the Lorenz curve, representing cumulative DALYs across the population ranked by SDI. Negative CI values indicate concentration of burden in low-SDI countries [26,27].

### 2.4 Statistical Analysis

DALYs estimates are reported with 95% UIs [24]. Percentage changes were calculated as [(2021 − 1990)/1990] × 100%. SII and CI standard errors were obtained from weighted regression and the delta method, respectively [27]. *p*-values < 0.05 (two-sided) were considered significant; non-overlapping 95% UIs between groups were considered indicative of significant differences, consistent with GBD practices [24]. GBD 2021 provides complete modeled estimates for all 204 countries (1990–2021) because its modeling framework (DisMod-MR 2.1, spatiotemporal Gaussian process regression) internally handles missing input data. Thus, the extracted variables had no missing values and required no imputation [24].

To assess robustness, we conducted two sensitivity analyses (**Supplementary Table 1**). First, we performed a leave-one-out analysis excluding Greenland—the territory with the highest burden—and recalculated the SII for 2021. Second, we recalculated the CI using two alternative SDI stratifications (tertiles and median split) and compared the results to the main quintile-based analysis.

We performed all analyses using R version 4.3.3 (R Foundation for Statistical Computing, Vienna, Austria, https://www.r-project.org/). Key packages included tidyverse and dplyr for data manipulation; data.table and tidytable for large datasets; ggplot2, patchwork, lemon, and ggpubr for plotting; epitools, rlang, ggsci, FactoMineR, and easyGBDR for GBD data processing, country-level calculations, and mapping; and car, MASS, mgcv, splines, broom, ggbrace, and easyGBDR for regression diagnostics, robust modeling, Lorenz curve fitting, inequality metrics, and annotated visualizations. This workflow enabled streamlined quantification and visualization of global mental health burdens and inequalities. GBD 2021 data are publicly accessible via the GBD Results Tool, and the analysis code is available from the corresponding author upon request.

## 3. Results

### 3.1 Global Burden of Mental Disorders

We grouped the ten mental disorder subtypes into five clinically meaningful categories: (1) mood disorders (depressive disorders and bipolar disorder); (2) anxiety and eating disorders (anxiety disorders and eating disorders); (3) schizophrenia; (4) neurodevelopmental disorders (autism spectrum disorders, ADHD, IDID and conduct disorder); and (5) other mental disorders.

Data from the GBD 2021 database indicated that mental disorders affected an estimated 1095.4 million individuals globally in 2021 (95% UI: 1016.5–1184.5), with a corresponding DALYs burden of 155.4 million (95% UI: 117.0–198.2), accounting for 5.39% (95% UI: 4.44%–6.30%) of the total burden across 371 diseases and representing a 73.4% increase relative to 1990 (89.6 million, 95% UI: 67.6–114.1). Among the five clinical groups, mood disorders—driven primarily by depressive disorders (89.6% increase)—exhibited the largest absolute DALYs increase. Anxiety disorders (84.8%) and eating disorders (61.9%) also rose substantially. Within neurodevelopmental disorders, autism spectrum disorders (45.6%) and conduct disorder (25.0%) increased, whereas ADHD (11.1%) and IDID (15.2%) had more modest changes. The largest rises in crude DALYs rates occurred for depressive (28.3%), anxiety disorders (24.9%), and other mental disorders (19.6%). For age-standardized rates, the most notable increases were for anxiety (18.2%), eating disorders (16.4%) and depressive disorders (13.4%) (Table 1).

**Table 1.** **Global burden of mental disorders in 1990 and 2021**.

	DALYs			Crude DALYs rates			Age-standardized DALYs rates		
	1990 (million) *	2021 (million)*	Change (%)	1990 (/100,000) *	2021 (/100,000) *	Change (%)	1990 (/100,000) *	2021 (/100,000) *	Change (%)
Mental disorders	89.6 (67.6–114.1)	155.4 (117.0–198.2)	73.4	1679.3 (1267.5–2139.7)	1969.5 (1483.0–2511.1)	17.3	1745.2 (1314.3–2214.5)	1909.1 (1440.2–2437.9)	9.4
Depressive disorders	29.7 (20.7–40.2)	56.3 (39.3–76.5)	89.6	556.4 (389.0–753.8)	713.8 (498.5–969.9)	28.3	600.5 (420.9–818.4)	681.1 (475.2–923.8)	13.4
Bipolar disorder	4.9 (3.2–7.1)	8.0 (5.2–11.5)	63.3	92.4 (60.5–133.2)	101.5 (65.7–145.7)	9.8	97.0 (63.4–139.5)	97.3 (63.1–140.0)	0.3
Anxiety disorders	23.0 (15.8–31.5)	42.5 (29.4–57.7)	84.8	431.2 (296.5–591.5)	538.7 (372.5–731.6)	24.9	443.7 (306.2–603.3)	524.3 (363.1–716.3)	18.2
Eating disorders	2.1 (1.3–3.4)	3.4 (2.1–5.4)	61.9	40.1 (24.2–63.5)	43.1 (26.3–68.0)	7.5	37.3 (22.7–58.6)	43.4 (26.3–68.4)	16.4
Schizophrenia	8.8 (6.5–11.3)	14.8 (10.9–19.1)	68.2	164.3 (121.4–211.2)	187.8 (138.5–242.0)	14.3	176.6 (130.9–226.0)	177.8 (131.5–228.8)	0.7
Autism spectrum disorders	7.9 (5.4–11.1)	11.5 (7.8–16.3)	45.6	147.5 (100.3–207.5)	146.3 (99.4–206.4)	–0.8	144.5 (98.3–203.3)	147.6 (100.2–208.2)	2.1
ADHD	0.9 (0.5–1.4)	1.0 (0.6–1.7)	11.1	16.3 (8.9–26.7)	13.1 (7.2–21.2)	–19.6	14.9 (8.1–24.5)	13.5 (7.4–21.9)	–9.4
IDID	3.3 (1.5–5.6)	3.8 (1.8–6.5)	15.2	61.5 (28.6–105.5)	48.3 (22.3–82.6)	–21.5	57.8 (26.8–99.5)	49.9 (23.2–85.3)	–13.7
Conduct disorder	4.0 (2.2–6.2)	5.0 (2.7–7.8)	25.0	74.6 (40.8–116.5)	63.4 (34.4–98.8)	–15.0	65.7 (35.9–102.7)	67.7 (36.8–105.5)	3.0
Other mental disorders	5.1 (3.3–7.6)	9.0 (5.7–13.5)	76.5	95.0 (61.0–142.6)	113.6 (72.5–170.9)	19.6	107.0 (68.6–161.3)	106.6 (68.2–160.7)	–0.4

*Central estimates with 95% uncertainty intervals. Percentages (%) were calculated by rounding the original estimates to one decimal place. Abbreviations: DALYs, disability-adjusted life-years; IDID, idiopathic developmental intellectual disability; ADHD, attention-deficit/hyperactivity disorder.

### 3.2 Age- and Sex-Stratified Global Burden of Mental Disorders

**Fig. 1.**
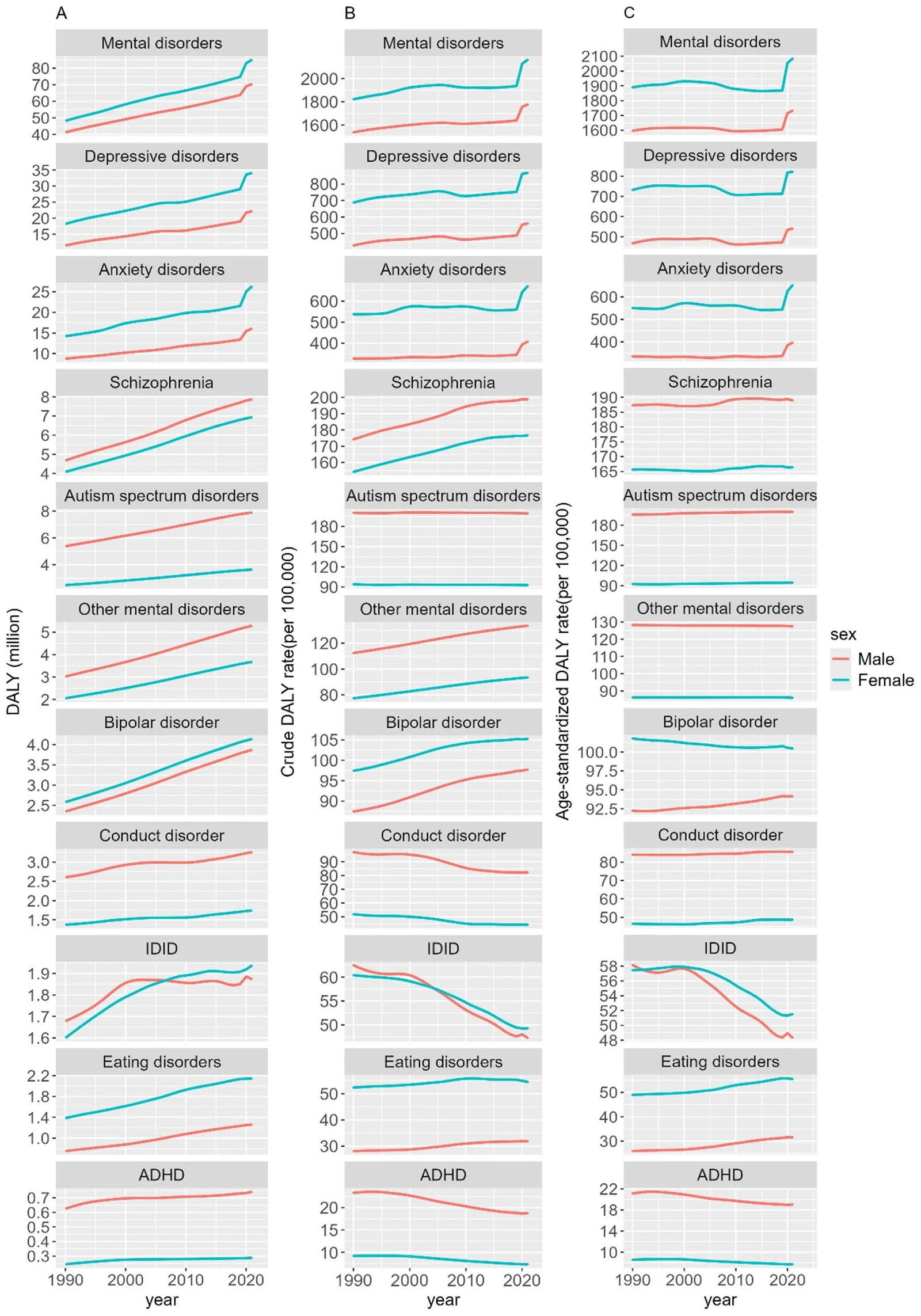
**Global burden of mental disorders from 1990 to 2021 stratified by sex**. (A) DALYs. (B) Crude DALY rates. (C) Age-standardized DALYs rate.

From 1990 to 2021, females consistently had a higher burden of mental disorders than males, even after age adjustment. This disparity was particularly pronounced for depressive disorders, anxiety disorders, bipolar disorder, and eating disorders. In contrast, males had a higher burden of schizophrenia, autism spectrum disorders, conduct disorder, ADHD, and other mental disorders (Fig. 1).

**Fig. 2.**
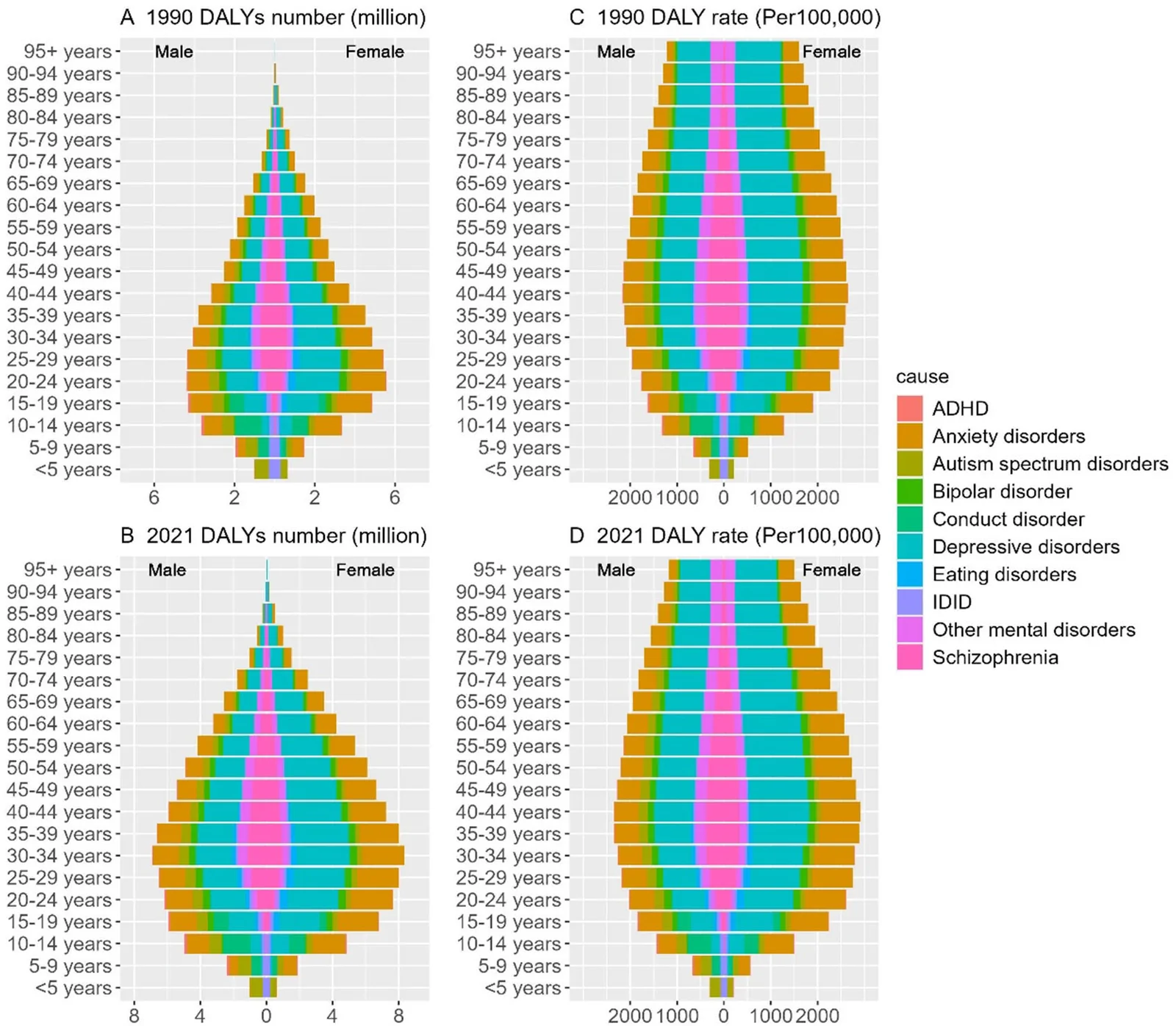
**Age distribution of mental disorder burden by sex in 1990 and 2021**. (A) DALYs (1990). (B) DALYs (2021). (C) DALYs rates (1990). (D) DALYs rates (2021).

From 1990 to 2021, the number of DALYs attributable to mental disorders increased across most age groups, with the most pronounced growth observed among individuals aged 30–54 years. Although the overall DALYs rate remained relatively stable over the study period, slight increases were observed in adolescents and selected young- to middle-aged groups. Overall, females aged 15–34 years had slightly higher DALYs counts and rates than males. Anxiety and depressive disorders were the leading contributors to DALYs, with a particularly strong impact on females and adolescents. ADHD was more concentrated in children and adolescents, whereas autism spectrum disorders also represented a notable burden in younger populations. Collectively, the burden of mental disorders increased substantially from 1990 to 2021, with the greatest impact being observed among young and middle-aged adults (Fig. 2).

### 3.3 Distribution of Mental Disorders Across Countries and Territories

**Fig. 3.**
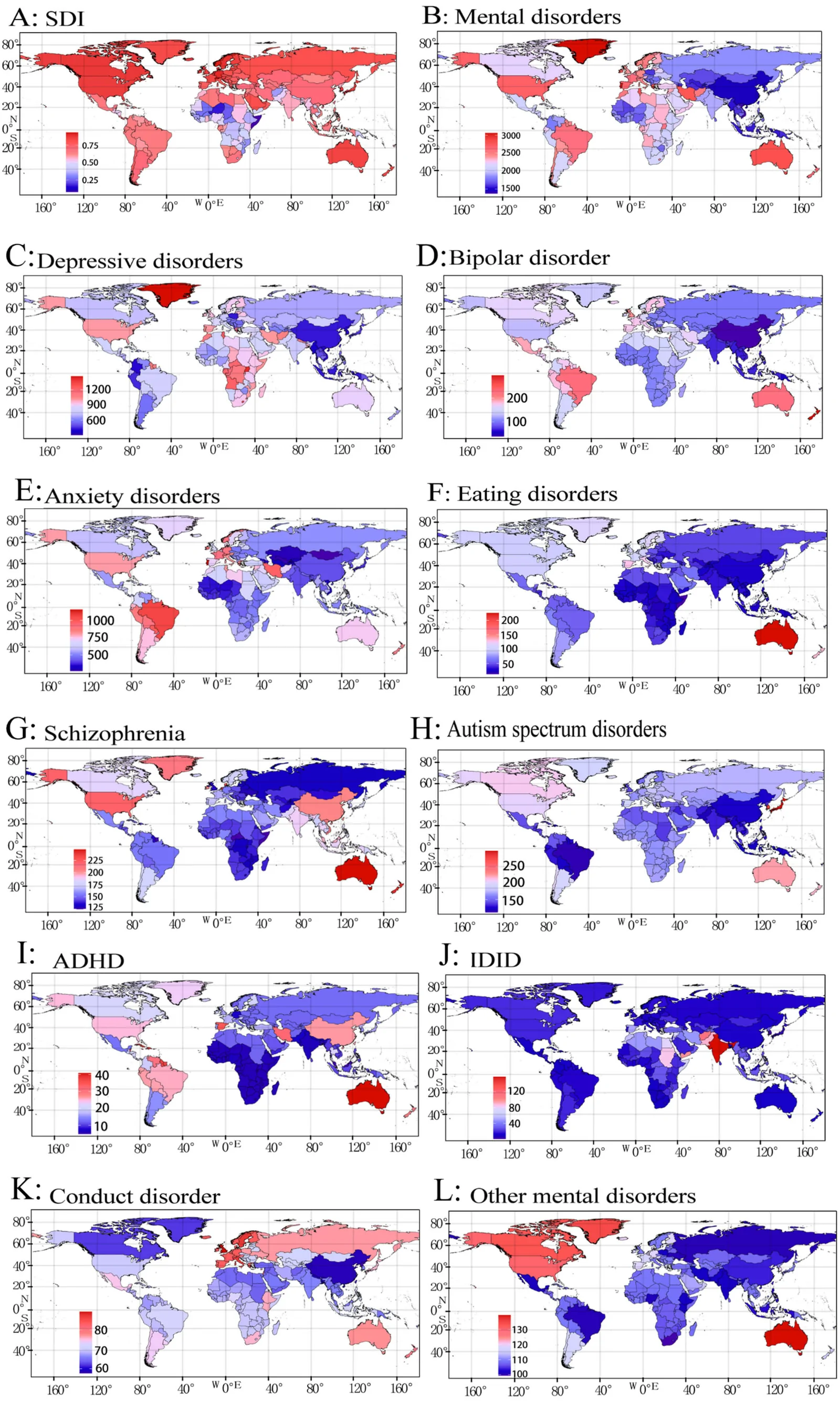
**Global spatial distribution of SDI and DALY rates of mental disorders in 2021**. (A) Socio-demographic Index (SDI) values across all countries and territories. (B–L) Spatial distribution of age-standardized DALYs rates for overall mental disorders and 10 specific mental disorder types across countries and territories.

The spatial distribution of age-standardized DALYs rates per 100,000 population for mental disorders and their ten subtypes in 2021 revealed substantial geographic variation. The overall burden of mental disorders was highest in Greenland and parts of North America, approaching 3000 per 100,000. Greenland, a high-SDI region, recorded the highest rate (3062.3), whereas Viet Nam, a middle-SDI region, had the lowest (1369.7). Disorder-specific patterns varied across regions. Depressive disorders were highest in Greenland (high SDI, 1467.9) and lowest in Brunei Darussalam (high-middle SDI, 306.3). Schizophrenia rates peaked in Australia (high SDI, 247.6) and were lowest in Somalia (low SDI, 124.8). Conduct disorder burden was highest in the United Kingdom (high SDI, 89.4) and the lowest in the Democratic People’s Republic of Korea (low-middle SDI, 57.6). ADHD levels were highest in Australia (high SDI, 40.8) and the lowest in Malaysia (high-middle SDI, 4.8). Anxiety disorders were most prevalent in Portugal (high-middle SDI, 1156.8) and least prevalent in Mongolia (low-middle SDI, 268.1). In contrast to the relatively geographically dispersed patterns observed for these five disorders, the remaining five exhibited more consistent and concentrated patterns. Other mental disorders were mainly observed in North America, Northern Europe (20° W–160° W, 20° N–80° N), and Australia, with the highest rate in Australia (high SDI, 139.5) and the lowest in South Africa (middle SDI, 99.4). IDID was largely concentrated in South Asia (60° E–90° E, 10° N–40° N), with India (low-middle SDI, 157.1) showing the highest burden and Singapore (high SDI, 0.6) the lowest. Eating disorders were predominantly observed in Australia, with the highest rate in Australia (high SDI, 225.9) and the lowest in Somalia (low SDI, 12.4). Bipolar disorder was primarily concentrated in South America (40° W–80° W, 0–40° S) and parts of Australia, with the highest rate in New Zealand (high SDI, 296.1) and the lowest in China (high-middle SDI, 37.7). Overall, these findings highlight substantial heterogeneity in age-standardized DALYs rates for mental disorders and their subtypes across different regions and SDI levels (Fig. 3, Table 2).

**Table 2.** **Age-standardized DALYs rates for overall mental disorders and 10 specific mental disorder types, showing the top 5 and bottom 5 countries/territories in 2021**.

	Mental disorders	Depressive disorders	Bipolar disorder	Anxiety disorders	Eating disorders	Schizophrenia	Autism spectrum disorders	ADHD	IDID	Conduct disorder	Other mental disorders
**Top 5 countries and territories**											
1	Greenland 3062.3 (2210.6–4093.9)	Greenland 1467.9 (965.0–2142.7)	New Zealand 296.1 (189.6–432.0)	Portugal 1156.8 (692.9–1723.8)	Australia 225.9 (145.8–330.9)	Australia 247.6 (186.2–300.2)	Japan 299.1 (207.2–420.0)	Australia 40.8 (23.0–63.2)	India 157.1 (83.0–249.7)	United Kingdom 89.4 (48.8–137.4)	Australia 139.5 (92.0–197.7)
2	Portugal 3038.2 (2167.0–3967.7)	Uganda 1419.6 (921.5–2059.5)	Brazil 229.9 (151.8–331.8)	Brazil 1058.7 (732.5–1442.3)	Monaco 193.3 (116.6–295.6)	New Zealand 243.4 (181.9–311.9)	Republic of Korea 283.8 (196.0–395.6)	Grenada 37.9 (20.6–61.4)	Afghanistan 112.2 (55.8–185.8)	Italy 87.4 (48.5–133.3)	Greenland 134.5 (87.4–197.3)
3	Lebanon 2905.4 (2075.8–3923.5)	Palestine 1375.1 (869.2–1998.3)	Australia 226.6 (145.1–334.3)	Paraguay 992.1 (637.3–1514.6)	New Zealand 132.7 (80.9–207.5)	Netherlands 233.6 (170.0–305.0)	Singapore 280.9 (193.9–388.0)	Cuba 37.7 (20.9–62.1)	Yemen 97.5 (47.1–162.6)	Sweden 87.4 (47.5–133.6)	Canada 133.0 (86.0–194.4)
4	Palestine 2880.8 (2030.6–3885.7)	Lesotho 1342.0 (880.5–1880.6)	Paraguay 216.4 (132.9–321.6)	Lebanon 981.0 (592.8–1442.8)	Luxembourg 132.6 (79.8–201.2)	Denmark 231.4 (173.0–282.7)	Brunei Darussalam 257.7 (176.3–361.1)	Dominica 37.6 (20.8–61.1)	Pakistan 89.6 (42.3–151.4)	Luxembourg 83.9 (45.8–132.8)	United States of America 130.8 (84.1–192.7)
5	Greece 2867.7 (2049.9–3860.5)	Tunisia 1255.1 (800.1–1861.4)	Israel 205.1 (123.1–300.2)	Iran (Islamic Republic of) 973.7 (659.1–1345.8)	Spain 127.6 (77.7–195.8)	United States of America 221.4 (164.6–281.5)	Australia 223.5 (155.0–315.0)	Saint Vincent and the Grenadines 37.6 (20.6–61.4)	Sudan 83.9 (40.2–142.7)	Israel 83.8 (45.3–132.7)	Singapore 120.4 (77.6–178.0)
**Bottom 5 countries and territories**											
1	Viet Nam 1369.7 (1031.6–1763.5)	Brunei Darussalam 306.3 (193.6–445.7)	China 37.7 (24.6–54.3)	Mongolia 268.1 (167.4–389.8)	Somalia 12.4 (7.3–19.6)	Somalia 124.8 (89.0–168.4)	Bangladesh 110.3 (75.4–154.6)	Malaysia 4.8 (2.6–8.4)	Singapore 0.6 (0.0–4.0)	Democratic People’s Republic of Korea 57.6 (31.6–90.7)	South Africa 99.4 (65.0–150.4)
2	Democratic People’s Republic of Korea 1440.6 (1073.7–1857.2)	Myanmar 387.3 (256.9–554.2)	Democratic People’s Republic of Korea 38.6 (23.1–58.5)	Uzbekistan 279.4 (169.2–447.4)	Democratic People’s Republic of Korea 16.7 (10.4–26.1)	Central African Republic 124.8 (85.1–171.3)	Brazil 114.3 (77.0–160.1)	United Arab Emirates 5.4 (3.0–8.9)	Brunei Darussalam 1.3 (0.0–6.1)	China 58.5 (32.2–91.7)	Russian Federation 100.5 (65.3–152.8)
3	China 1446.8 (1102.9–1826.7)	Taiwan (Province of China) 389.9 (249.8–542.9)	Taiwan (Province of China) 39.4 (23.4–58.1)	Kazakhstan 292.5 (187.2–431.6)	Kiribati 18.2 (11.2–28.7)	Suriname 125.5 (92.1–159.5)	Haiti 116.5 (79.5–164.7)	Gabon 5.8 (3.1–9.7)	Republic of Korea 1.3 (0.0–6.5)	Bangladesh 58.7 (32.0–91.3)	Brazil 100.6 (65.3–152.7)
4	Taiwan (Province of China) 1490.0 (1107.1–1937.6)	Singapore 398.3 (261.4–582.2)	Marshall Islands 55.0 (33.0–80.7)	Kyrgyzstan 304.5 (183.2–460.6)	Solomon Islands 18.4 (11.3–28.7)	Haiti 127.2 (90.2–173.5)	Nepal 116.9 (78.7–164.4)	Mozambique 5.8 (3.0–9.8)	Denmark 2.0 (0.1–5.6)	Nepal 58.8 (31.9–91.2)	Ukraine 100.6 (64.9–151.0)
5	American Samoa 1533.6 (1141.7–1992.7)	Viet Nam 414.6 (270.0–580.6)	Kiribati 55.2 (33.2–81.2)	Viet Nam 309.1 (195.7–476.3)	Burundi 19.0 (11.3–30.5)	Republic of Moldova 127.3 (88.4–173.1)	Paraguay 117.4 (79.0–161.8)	United Republic of Tanzania 5.9 (3.1–9.8)	Monaco 3.1 (0.2–8.5)	Bhutan 58.8 (32.6–92.5)	Nigeria 100.9 (65.5–154.2)

### 3.4 Absolute Inequality

**Fig. 4.**
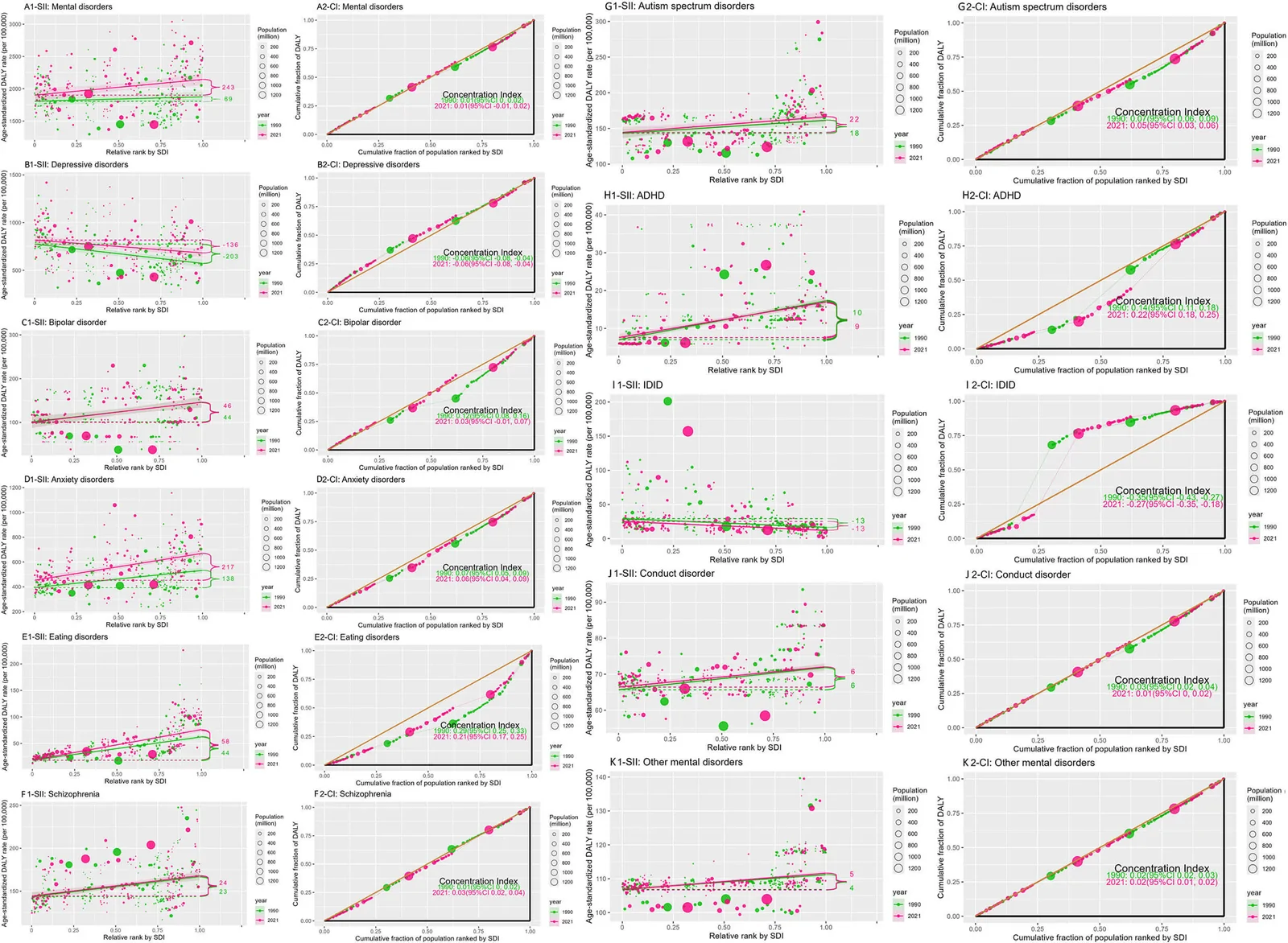
**SDI-related absolute and relative inequalities in mental disorder burden, 1990–2021**. (A1–K1) Absolute SDI-related health inequality in the burden of mental disorders, shown using regression lines for 1990 and 2021. Each dot represents a country or territory, with sizes proportional to population. (A2–K2) Relative SDI-related health inequality, illustrated by concentration (Lorenz) curves for 1990 and 2021. The x-axis shows the cumulative population ranked by national SDI, and the y-axis shows the cumulative fraction of age-standardized DALYs.

For absolute inequality measured by the SII, significant changes were observed from 1990 to 2021 across the five clinical groups (Fig. 4). Overall, the SII for mental disorders increased more than threefold, indicating that the absolute gap between high- and low-SDI countries has widened substantially. Among mood disorders, the SII for depressive disorders decreased from −203 to −136 (a negative SII indicates higher burden in low-SDI countries; the decrease in magnitude means the gap has narrowed). The SII for bipolar disorder increased from 44 to 46. Within anxiety and eating disorders, the SII increased for both anxiety disorders (138 to 217) and eating disorders (44 to 58), reflecting widening absolute gaps favoring high-SDI countries. For neurodevelopmental disorders, trends were mixed: SII increased for autism spectrum disorders (18 to 22), remained unchanged for conduct disorder (6), decreased for ADHD (10 to 9), and stayed stable for IDID (−13). The stable negative SII for IDID indicates a persistently higher burden in low-SDI countries. The SII for schizophrenia increased from 23 to 24, while other mental disorders rose from 4 to 5 (Fig. 4).

### 3.5 Relative Inequality

Regarding relative inequality measured by the CI, changes from 1990 to 2021 also varied across clinical groups (Fig. 4). Overall, the CI for all mental disorders remained stable at 0.01, suggesting no change in relative concentration. Among mood disorders, the CI for depressive disorders remained stable at −0.06, while bipolar disorder showed a decrease (0.12 to 0.03), indicating narrowing relative inequality. For anxiety and eating disorders, both CI values declined: anxiety disorders from 0.07 to 0.06, and eating disorders from 0.29 to 0.21, suggesting proportional convergence across SDI levels. For neurodevelopmental disorders, patterns diverged: the CI increased for ADHD (0.14 to 0.22) and decreased for autism spectrum disorders (0.07 to 0.05), conduct disorder (0.03 to 0.01), and IDID (−0.35 to −0.27). The negative CI for IDID confirms its persistent concentration in low-SDI countries, and the decrease indicates narrowing relative inequality. Schizophrenia increased from 0.01 to 0.03, indicating widening relative inequality, while other mental disorders remained stable at 0.02 (Fig. 4).

## 4. Discussion

In this study, we used GBD 2021 data to assess global trends and cross-country inequalities in the burden of ten mental disorder subtypes from 1990 to 2021. All findings represent associations at the population level and should not be interpreted as causal at the individual level. Three main findings emerged. First, the global burden of mental disorders increased substantially: total DALYs rose by 73.4% and age-standardized rates by 9.4%, with anxiety and eating disorders showing the fastest growth. Second, the burden was unequally distributed by sex. Females had higher rates of depressive, anxiety, bipolar, and eating disorders, whereas males had higher rates of schizophrenia, autism spectrum disorders, conduct disorder, and ADHD. Third, and most centrally, cross-national inequalities persisted or widened for most subtypes. The SII for overall mental disorders increased from 69 to 243, indicating widening absolute gaps between high- and low-SDI countries, while the CI remained stable at 0.01. Notably, high-SDI countries carried heavier burdens of eating disorders, ADHD, schizophrenia, and anxiety disorders, whereas low-SDI countries bore heavier burdens of IDID and depressive disorders. These findings are novel because they offer a combined assessment of both absolute and relative inequalities across all ten mental disorder subtypes over a 32-year period, revealing divergent trends that are not apparent from single-disorder or age-restricted analyses.

Zhang et al. [18] recently examined depressive and anxiety disorders in adolescents (aged 10–24 years) using GBD 2021 data, reporting that absolute inequality (SII) increased while relative inequality (CI) decreased. Our study extends their findings in three ways. First, by covering all ages, we identify that the burden increase is most pronounced among adults aged 30–54 years—a group not considered previously. Second, across ten disorder subtypes, we show that inequality patterns are not uniform. Unlike depression and anxiety in youth, schizophrenia and ADHD exhibit increasing inequality in both absolute and relative terms (schizophrenia: SII 23→24, CI 0.01→0.03; ADHD: CI 0.14→0.22). Third, for depressive disorders (all ages), absolute inequality narrowed (SII from −203 to −136), contrasting with the adolescent-only trend reported by Zhang et al. [18]. These disorder- and age-specific patterns underscore the need for disaggregated analyses in global mental health policy.

Beyond these overall patterns, inequality trends varied across specific disorders. Absolute inequality increased for six disorders (autism spectrum disorders, anxiety disorders, bipolar disorder, eating disorders, other mental disorders, and schizophrenia) but decreased for depressive disorders and ADHD. Relative inequality increased only for schizophrenia and ADHD, while decreasing for most other disorders. These divergent trends—widening absolute inequality alongside stable or narrowing relative inequality—highlight the importance of examining both dimensions when assessing global mental health disparities. As far as we are aware, such findings have not been reported before.

The SII and CI are standard tools for monitoring health inequalities across socioeconomic strata [26]. Unlike simpler indicators such as the range, they capture inequality across the entire SDI distribution rather than comparing only extreme groups [19]. The SII quantifies absolute inequality on the original DALYs scale, whereas the CI measures relative inequality—the proportional concentration of burden across the SDI distribution [19,28]. Widening absolute inequality signals increasing population health gaps, while narrowing relative inequality may indicate proportional convergence even as absolute gaps persist [28]. In this study, we report both metrics to provide a more complete evidence base for global mental health policy.

Our study found that the burden of mental disorders was generally higher in females than in males over the 32-year study period, particularly for depression and anxiety. This sex disparity is consistent with previous research showing higher prevalence and incidence of depression and anxiety disorders in females [29,30]. The female predominance may be explained by hormonal fluctuations across the reproductive life course and their interaction with the hypothalamic–pituitary–adrenal axis [31], as well as social determinants such as lower social status [32,33] and longer life expectancy [34]. In contrast, males bore a higher burden of conduct disorder and autism spectrum disorder, consistent with previous reports of male predominance in these conditions [35,36]. Together, these findings suggest that while the mental health burden in females warrants widespread attention, developmental mental health issues in males should not be overlooked.

Geographically, the highest burden of mental disorders in 2021 was concentrated in Greenland and parts of North America, possibly related to extreme seasonal variations in daylight exposure. These regions experience significant differences in daylight hours due to seasonal changes, with very short daylight periods from September to March and continuous sunlight during summer, resulting in the midnight sun phenomenon [37,38]. Greater night-time light exposure has been demonstrated to be associated with an increased risk of major depressive disorder, generalized anxiety disorder, post-traumatic stress disorder (PTSD), psychosis, bipolar disorder, and self-harm. Greater daytime light exposure is also independently associated with a reduced risk of major depressive disorder, PTSD, psychosis, and self-harm [39].

Economic factors critically shape the global distribution of mental disorder burden [40]. A bidirectional causal relationship exists between poverty and common mental illnesses—particularly depression and anxiety—creating a self-reinforcing poverty trap [41]. Mechanisms include chronic stress, housing and food insecurity, environmental hazards, and early-life deprivation, which impair cognitive development and increase lifelong vulnerability [42]. Our results show that from 1990 to 2021, the SII for overall mental disorders increased from 69 to 243 (widening absolute inequality), whereas the CI remained stable at 0.01 (no change in relative concentration). This divergence is consistent with a scenario where high- and low-SDI countries experienced similar proportional increases in burden, but the absolute gap widened because high-SDI countries started from a higher baseline [43]. In other words, while relative inequality remained unchanged, the absolute health gap between the richest and poorest countries has grown substantially. However, this interpretation is inferential, as we did not directly test for statistical equivalence in proportional increases across SDI groups. A more definitive assessment would require examining trends within each SDI quintile [1]. This pattern—proportional improvements (stable CI) coexist with widening absolute gaps—is analogous to the Easterlin paradox, which observes that economic growth at the national level does not always translate into proportional increases in individual well-being [44]. In both cases, aggregate improvements may mask persistent or widening absolute disparities between subgroups.

In 2021, high-SDI countries experienced a higher burden of eating disorders, ADHD, schizophrenia, and anxiety disorders. These four disorders are characterized by chronic, relapsing courses requiring long-term specialized care [45,46,47], placing substantial demands on health systems even in high-resource settings. Conversely, low-SDI countries exhibited higher burdens of depressive disorders and IDID. The higher burden of certain disorders in high-SDI countries may partly reflect differences in diagnostic practices, healthcare access, and reporting systems rather than true differences in disease occurrence [15,24]. High-SDI countries have more developed mental health infrastructure and lower stigma, leading to higher case ascertainment, whereas underdiagnosis of disorders requiring specialized assessment (e.g., ADHD, autism) is common in low-SDI settings [15]. However, detection bias cannot fully explain all patterns: depressive disorders and IDID showed higher burden in low-SDI countries, consistent with etiological expectations. Readers should interpret SDI-stratified findings with awareness of these asymmetries. Despite higher reported burdens in high-SDI countries, global mental health spending remains grossly inadequate, particularly in low- and middle-income countries, where per capita expenditure is less than $2 compared to over $50 in wealthy countries [48]. On average, only 3% of government health budgets are allocated to mental health, ranging from <1% in low-income countries to 5% in high-income countries. Even at 5%, nearly 50% of patients with depression do not receive treatment [49]. These financing gaps are likely to exacerbate existing inequalities in mental disorder burden across SDI levels. Our study also revealed that the burden of IDID is concentrated in low SDI countries. Intellectual disability (ID) can be caused by environmental or genetic factors [50]. While the etiology of non-genetic ID is not fully understood, Huang et al. [51] proposed a set of 10 prenatal factors, one perinatal factor (prematurity), and two neonatal factors (male sex and low birth weight) that are significantly associated with an increased risk of ID. Additionally, McDermott et al. [52] found that IDID is associated with soil and water concentrations of heavy metals such as arsenic and lead. In India, levels of arsenic, cadmium, and copper exceed established limits in all soil types except roadside soil [53]. The prevalence of conduct disorder is closely related to family relationships [54], genetic factors [55], and neighborhood effects [56].

The GBD 2021 estimates cover 1990 to 2021, thereby including the COVID-19 pandemic years 2020–2021. GBD 2021 incorporated available survey data from this period and adjusted its models for depressive and anxiety disorders to reflect pandemic-attributable increases [20,24]. However, data availability varied substantially across countries during the pandemic, leading to heterogeneous adjustment quality. Thus, the pronounced increases observed for depressive and anxiety disorders—particularly in the final two years of the study period—should be interpreted with the understanding that they may partially capture pandemic effects rather than representing a simple continuation of pre-2020 trends.

Promoting mental health is a global public good and is essential for sustainable development in all countries, regardless of socioeconomic status, as every country can be considered “developing” in the context of mental health [1]. According to the World Health Organization’s latest *Mental Health Atlas*, most of the global mental health targets set for 2020 were not achieved, and the field continues to suffer from significant underinvestment [57]. Moreover, levels of inequality in terms of the burden of mental disorders across SDI levels have generally widened over the past three decades, suggesting that improvements in sociodemographic conditions have not been matched by adequate investments in the prevention, management, and treatment of these disorders, particularly in low- and middle-income countries. Based on these findings, we propose three policy actions. First, given the widening absolute inequality alongside stable relative inequality, global health financing should incorporate redistributive mechanisms, such as targeted resource allocation from high- to low-SDI countries via WHO Mental Health Gap Action Programme. Second, low-SDI countries, which bear higher burdens of depressive disorders and IDID, should prioritize scaling up community-delivered depression interventions and investing in early-life environmental health to reduce known risk factors. Third, high-SDI countries, facing greater burdens of eating disorders, ADHD, schizophrenia, and anxiety disorders, need to strengthen specialized long-term care pathways given the chronic nature of these conditions. Failure to implement these equity-oriented policies will likely worsen absolute disparities between the world’s richest and poorest nations.

### Limitations

This study employed SII and CI values to systematically assess inequality in the burden of mental disorders. However, several limitations should be acknowledged. First, our study is limited by the varying quality of GBD data and by missing data, especially from underdeveloped countries. Second, the global burden of mental disorders may be underestimated, which could bias our results. Third, the SII and CI were calculated only for 1990 and 2021. While this two-time-point approach captures net long-term change, it does not capture non-monotonic patterns or fluctuations over the intervening 32 years. Because we extracted annual data, the restriction to two endpoints necessarily entails a loss of non-linear information. Future work should compute these inequality metrics at annual or 5-year intervals (e.g., GBD 2023) to reveal continuous trajectories and better inform policy. Fourth, our analysis is based on GBD 2021, as GBD 2023 was released after the completion of this study. Future research using updated GBD data is warranted to validate our findings and assess the robustness of the observed trends and inequalities across different GBD iterations.

## 5. Conclusions

In conclusion, from 1990 to 2021, age-standardized DALYs rates for mental disorders showed an upward trend, driven by anxiety, eating, and depressive disorders, particularly among females and adults aged 30–54 years. More importantly, absolute cross-national inequality in mental disorder burden widened for most disorder subtypes, with the SII for overall mental disorders increasing from 69 to 243. However, the distribution of burden across SDI strata is disorder-specific: high-SDI countries bear heavier burdens of eating disorders, ADHD, schizophrenia, and anxiety disorders, whereas low-SDI countries bear heavier burdens of depressive disorders and IDID. Critically, absolute and relative inequality can diverge—widening absolute gaps alongside stable or narrowing relative inequality—highlighting that proportional convergence does not reduce absolute disparities. To address these persistent inequalities, global mental health policies must prioritize anxiety and depressive disorders while tailoring interventions to the distinct epidemiological profiles of high- versus low-SDI countries. Without targeted resource allocation to low-SDI countries, the mental health gap between the world’s richest and poorest nations will likely continue to widen.

## Data Availability

The data used in this study are publicly available from the Global Burden of Disease 2021 Study. All data can be accessed via the GBD Results Tool (https://gbd2021.healthdata.org/gbd-results/). The analysis code is available from the corresponding author upon reasonable request.

## Abbreviations

ADHD, attention-deficit/hyperactivity disorder; CI, concentration index; DALYs, disability-adjusted life years; DSM-IV, diagnostic and statistical manual of mental disorders, fourth edition; GBD, Global Burden of Diseases; ICD-10, international classification of diseases, 10th revision; ID, intellectual disability; IDID, idiopathic developmental intellectual disability; PTSD, post-traumatic stress disorder; SDI, socio-demographic index; SII, slope index of inequality; UI, uncertainty intervals.
