# Supplementary material for: Global Burden and Inequalities in Mental Disorders Across Countries and Territories: 1990 to 2021
Source: Alpha Psychiatry. 2026 Aug 13;27(4):51756. doi: 10.31083/AP51756 (PMC13540006; doi:10.31083/AP51756)
Supplement: Supplementary file 1 [file 2757-8038-27-4-51756-s1.zip › Supplementary Table 1.pdf]

Supplementary Table S1. Sensitivity analyses for outlier exclusion (excluding Greenland) and alternative SDI stratification schemes

Disorder	Main SII (2021)	SII without Greenland	Relative change (%)	Main CI (quintiles)	CI (tertiles)	CI (median split)	CI (exclude middle tertile)
Mental disorders	243	233	-4.0	0.01 (-0.01 to 0.03)	0.01 (-0.01 to 0.03)	0.01 (-0.01 to 0.04)	0.02 (-0.01 to 0.05)
Depressive disorders	-136	-141	+3.5	-0.06 (-0.09 to -0.03)	-0.06 (-0.09 to -0.03)	-0.07 (-0.10 to -0.03)	-0.07 (-0.11 to -0.03)
Bipolar disorder	46	46	-0.4	0.03 (0.00 to 0.06)	0.03 (0.00 to 0.06)	0.03 (-0.01 to 0.07)	0.04 (0.00 to 0.08)
Anxiety disorders	217	216	-0.6	0.06 (0.03 to 0.09)	0.06 (0.03 to 0.09)	0.06 (0.02 to 0.10)	0.07 (0.03 to 0.11)
Eating disorders	58	57	-2.2	0.21 (0.15 to 0.27)	0.21 (0.14 to 0.28)	0.20 (0.13 to 0.27)	0.22 (0.14 to 0.30)
Schizophrenia	24	23	-3.1	0.03 (0.01 to 0.05)	0.03 (0.01 to 0.05)	0.03 (0.00 to 0.06)	0.04 (0.01 to 0.07)
Autism spectrum disorders	22	22	+2.2	0.05 (0.02 to 0.08)	0.05 (0.02 to 0.08)	0.05 (0.01 to 0.09)	0.06 (0.02 to 0.10)

Disorder	Main SII (2021)	SII without Greenland	Relative change (%)	Main CI (quintiles)	CI (tertiles)	CI (median split)	CI (exclude middle tertile)
ADHD	9	9	+3.1	0.22 (0.16 to 0.28)	0.22 (0.15 to 0.29)	0.21 (0.14 to 0.28)	0.23 (0.15 to 0.31)
IDID	-13	-13	+1.8	-0.35 (-0.42 to -0.28)	-0.35 (-0.43 to -0.27)	-0.36 (-0.44 to -0.28)	-0.38 (-0.47 to -0.29)
Conduct disorder	6	6	-2.8	0.01 (-0.02 to 0.04)	0.01 (-0.02 to 0.04)	0.01 (-0.03 to 0.05)	0.02 (-0.02 to 0.06)
Other mental disorders	5	5	-4.1	0.02 (-0.01 to 0.05)	0.02 (-0.01 to 0.05)	0.02 (-0.02 to 0.06)	0.03 (-0.01 to 0.07)

Notes: SII = slope index of inequality (absolute inequality); CI = concentration index (relative inequality); UI = uncertainty interval; IDID = idiopathic developmental intellectual disability; ADHD = attention-deficit/hyperactivity disorder. Relative change calculated as (SII_without_Greenland - SII_main) / |SII_main| × 100%. All CIs rounded to two decimal places; UI bounds rounded to two decimal places. "Exclude middle tertile" refers to analysis excluding countries in the middle SDI tertile (SDI between 0.45 and 0.75), retaining only the highest (n=68) and lowest (n=68) SDI countries.
